# Kinetic modeling of ^68^Ga-PSMA-11 and validation of simplified methods for quantification in primary prostate cancer patients

**DOI:** 10.1186/s13550-020-0594-6

**Published:** 2020-02-24

**Authors:** Anna Ringheim, Guilherme de Carvalho Campos Neto, Udunna Anazodo, Lumeng Cui, Marcelo Livorsi da Cunha, Taise Vitor, Karine Minaif Martins, Ana Cláudia Camargo Miranda, Marycel Figols de Barboza, Leonardo Lima Fuscaldi, Gustavo Caserta Lemos, José Roberto Colombo Junior, Ronaldo Hueb Baroni

**Affiliations:** 1 Hospital Israelita Albert Einstein, Avenida Albert Einstein 627/701, Morumbi, Sao Paulo, SP CEP 05652-900 Brazil; 2 Lawson Health Research Institute, St Joseph’s Health Care, 268 Grosvenor Street, London, Ontario N6A 4V2 Canada; 3 Department of Medical Biophysics, Western University, 1151 Richmond Street N, London, Ontario N6A 5C1 Canada; 4 Division of Biomedical Engineering, University of Saskatchewan, 57 Campus Drive, Saskatoon, Saskatchewan, SK S7N 5A9 Canada

**Keywords:** Prostate cancer, PSMA, PET/MR, Kinetic modeling, Arterial blood sampling, Image-derived input function, SUV

## Abstract

**Background:**

The positron emission tomography (PET) ligand ^68^Ga-Glu-urea-Lys(Ahx)-HBED-CC (^68^Ga-PSMA-11) targets the prostate-specific membrane antigen (PSMA), upregulated in prostate cancer cells. Although ^68^Ga-PSMA-11 PET is widely used in research and clinical practice, full kinetic modeling has not yet been reported nor have simplified methods for quantification been validated. The aims of our study were to quantify ^68^Ga-PSMA-11 uptake in primary prostate cancer patients using compartmental modeling with arterial blood sampling and to validate the use of standardized uptake values (SUV) and image-derived blood for quantification.

**Results:**

Fifteen patients with histologically proven primary prostate cancer underwent a 60-min dynamic ^68^Ga-PSMA-11 PET scan of the pelvis with axial T1 Dixon, T2, and diffusion-weighted magnetic resonance (MR) images acquired simultaneously. Time-activity curves were derived from volumes of interest in lesions, normal prostate, and muscle, and mean SUV calculated. In total, 18 positive lesions were identified on both PET and MR. Arterial blood activity was measured by automatic arterial blood sampling and manual blood samples were collected for plasma-to-blood ratio correction and for metabolite analysis. The analysis showed that ^68^Ga-PSMA-11 was stable in vivo. Based on the Akaike information criterion, ^68^Ga-PSMA-11 kinetics were best described by an irreversible two-tissue compartment model. The rate constants *K*_1_ and *k*_3_ and the net influx rate constants *K*_i_ were all significantly higher in lesions compared to normal tissue (*p* < 0.05). *K*_i_ derived using image-derived blood from an MR-guided method showed excellent agreement with *K*_i_ derived using arterial blood sampling (intraclass correlation coefficient = 0.99). SUV correlated significantly with K_i_ with the strongest correlation of scan time-window 30–45 min (rho 0.95, *p* < 0.001). Both *K*_i_ and SUV correlated significantly with serum prostate specific antigen (PSA) level and PSA density.

**Conclusions:**

^68^Ga-PSMA-11 kinetics can be described by an irreversible two-tissue compartment model. An MR-guided method for image-derived blood provides a non-invasive alternative to blood sampling for kinetic modeling studies. SUV showed strong correlation with *K*_i_ and can be used in routine clinical settings to quantify ^68^Ga-PSMA-11 uptake.

## Background

The positron emission tomography (PET) ligand ^68^Ga-Glu-urea-Lys(Ahx)-HBED-CC (^68^Ga-PSMA-11) has dramatically improved diagnostic imaging of prostate cancer. PSMA-11 targets the prostate-specific membrane antigen (PSMA), which is overexpressed in most prostate cancer cells [1]. Several studies have shown that the combination of ^68^Ga-PSMA-11 PET with anatomical information from magnetic resonance (MR) imaging provides superior detection and diagnostic accuracy of prostate cancers in comparison to other imaging techniques [2–6].

In routine clinical settings, the most commonly used method for PET quantification is the standardized uptake value (SUV). Its calculation is simple and routinely available in commercial imaging software packages. SUV is a semi-quantitative measure of the total activity concentration in a region-of-interest at a time point of the PET scan, normalized by the injected activity and the subject’s weight. Studies have shown that SUV may differ between sites, when not controling for image acquisition parameters such as scan time post-tracer injection, reconstruction algorithms, and attenuation and scatter corrections [7]. For accurate quantification of PET tracer uptake, pharmacokinetic compartmental modeling with dynamic scanning and arterial blood sampling is usually necessary. However, due to the long and complex acquisition protocols, simplified acquisition and analysis methods are adopted in clinical practice. Their correlation to compartmental modeling should however first be assessed [7].

Methods to extract image-derived blood information provide an attractive alternative to arterial blood sampling, as they noninvasively obtain the arterial blood curve during the PET scan. For whole-body PET, most methods outline regions-of-interest (ROIs) over the common iliac artery of the PET image to obtain the blood data. These methods, however, usually suffer from partial volume effects due to limited spatial resolution of the PET image. With combined PET/MR, the high-resolution MR image can be used to outline the arteries and when combined with partial volume correction approaches [8], provide a more accurate blood input function for kinetic modeling [9].

Previously published studies on the biodistribution and kinetics of ^68^Ga-PSMA-11 in prostate cancer patients reported increasing SUV in tumor lesions from early to late imaging and rapid blood clearance of ^68^Ga-PSMA-11 [10–12]. SUV correlated strongly with the net influx rate *K*_i_, moderately with serum prostate-specific antigen (PSA) and weakly with Gleason score from biopsy [11, 12]. The authors used PET image-derived blood as input to the compartment model analysis, and omitted correction for plasma-to-blood ratio and study of labeled metabolites in plasma. To date, full kinetic modeling with arterial blood sampling and metabolite analysis has not yet been reported, nor has the use of simplified methods as SUV and image-derived blood data been validated.

Therefore, the objectives of the present study were to evaluate the kinetics of ^68^Ga-PSMA-11 using dynamic PET/MR scanning and arterial blood sampling, to validate the use of simplified methods for quantification and to correlate the results to patient, MR, and histopathological data.

## Methods

### Patients

Fifteen patients were included in this observational prospective study. The study was approved by the institutional ethics committee and informed consent was obtained from all individual participants included in the study. Inclusion criteria were histologically proven prostate cancer (Gleason score ≥ 6), with a tumour size of at least 0.6 cm in diameter on MR imaging. Only patients considered able to remain supine for at least one hour were included. Exclusion criteria were multiple malignancies, anticoagulant therapy and contraindications to MR imaging, such as claustrophobia and MR-incompatible implants. Serum PSA level had been measured previously and the prostate weight had been estimated by a previous MR scan. PSA density was calculated as serum PSA divided by the prostate weight.

### PET/MR protocol

The patients underwent a 60-min dynamic PET scan in list-mode with the field of view over the pelvic area with arms down on a Biograph mMR scanner (Siemens, Germany), followed by a whole-body PET/MR scan for clinical purposes. At the start of the PET scan, a bolus injection of ^68^Ga-PSMA-11 (median 191.6 MBq, range 103.9–231.3 MBq) was administered intravenously and flushed with 40 mL of saline. ^68^Ga-PSMA-11 was synthesized according to Eder et al. [13].

We used a 24-channel spine radiofrequency coil integrated within the MR bed and three surface body coils (6 channels each) to cover the thorax, abdomen, and pelvis. The following MR data were acquired during the PET scan: axial T1 volumetric interpolated breath-hold examination (VIBE) Dixon (18 s), axial T2 half-Fourier single shot turbo spin echo (HASTE) without fat suppression with breath-holding (23 s) and axial diffusion-weighted imaging without breath-holding (2 min 19 s). PET data were reconstructed with a 3D ordered-subsets expectation maximization (OSEM) algorithm (3 iteration, 21 subsets, matrix 256 × 256, 4 mm Gaussian filter) and corrected for decay, scatter, and attenuation using Dixon-based MR sequences. The list mode data were reconstructed into 28 frames (10 × 30 s, 5 × 60 s, 5 × 120 s, and 8 × 300 s).

### Image analysis

The reconstructed PET/MR images were analyzed by a board-certified nuclear medicine physician and a radiologist using the software syngo.via (Siemens, Germany). The analysis included visual assessment of the number and types of lesions. Lesion TACs in unit Bq/mL were derived from volumes-of-interest (VOIs) using isocontour threshold of 40% of maximum SUV on late PET images (last 15 min of scan) with the location confirmed on the T2-weighted MR images. Spherical VOIs of 1 mL were outlined on normal prostate and gluteus muscle to derive normal tissue TACs. MR images were analyzed using the Prostate Imaging Reporting and Data System version 2 (PI-RADS v2) [14] and the apparent diffusion coefficients (ADC) and lesion sizes were measured.

SUV in unit g/mL was calculated using the following formula: SUV = tissue concentration (kBq/mL)/(injected dose (MBq)/subject’s body weight (kg)). Mean SUV, which is the average SUV within a volume, was calculated for all VOIs.

### Arterial input function

Arterial blood activity was measured by continuous blood sampling from the radial artery during the first 10 min using an automatic blood sampling device with 1 s temporal resolution (Twilite, Swisstrace GmbH, Switzerland). The blood sampling device was connected to a shielded pump (B Braun Infusomat Space), using flow rates 4.17 mL/min for the first 7 min, then 2.5 mL/min for the remaining 3 min. The blood sampling device had been calibrated previously to the PET/MR scanner. Manual arterial blood samples (4.9 mL) were collected in heparinized tubes at 6 time points (approximately 3, 7, 15, 25, 40, and 60 min post-injection), while the automatic blood sampling was briefly paused.

The manual blood samples were immediately put on ice and centrifuged to separate plasma. Whole blood and plasma-activities were measured in a gamma counter (Triathler, HIDEX, Finland), which had been calibrated previously to the PET/MR scanner.

The arterial input function (AIF) was generated from the blood sampler curve corrected for decay, background, and dispersion, and resampled using the PSAMPLE software (PMOD, Switzerland). Using an in-house MATLAB program, the AIF was merged with the decay-corrected manual whole blood samples to get a 60-min AIF. This whole blood AIF was then converted into a plasma AIF using the average plasma-to-blood activity ratios from the manual samples for each subject.

### Metabolite analysis

The plasma samples were analyzed for possible labeled metabolites using an ultra-high-pressure liquid chromatography (UHPLC) method. The samples were filtered by centrifugation at 20,000×*g* for 10 min at 4 °C using a filter with a molecular weight cut-off of 3 kDa and analyzed on a 1290 Infinity II UHPLC system (Agilent Technologies, USA) equipped with a radioactivity detector (Eckert & Ziegler, Germany). A volume of 100 μL was loaded using automatic injection and the samples were maintained at 4 °C. The analytical column was a Phenomenex Kinetex® reverse-phase C18 column (100 mm × 3 mm; 2.6 μm), maintained at 25 °C with water + 0.1% trifluoroacetic acid (TFA) (mobile phase A; 95%) and acetonitrile + 0.1% TFA (mobile phase B; 5%) at flow rate 0.8 mL/min. Gradient of mobile phase B was 5–10% (0.0–0.5 min); 10–30% (0.5–7.0 min); 30–5% (7.0–7.5 min); 5% (7.5–15.0 min). Due to the low activity concentration, UHPLC fractions were collected every 20 s and counted in an automatic gamma counter (Wizard 2TM 3” 2480, Perkin Elmer, USA). Measured activity was plotted as a function of time and compared to the retention time of a standard solution containing ^68^Ga-PSMA-11, analyzed using the same chromatographic protocol. To determine its stability in vitro, an aliquot of 19 MBq of ^68^Ga-PSMA-11 was incubated in 1 mL of plasma for 60 min at 37 °C and analyzed using the same protocol.

### Image-derived input function

The image-derived input functions (IDIFs) were generated by extracting the median PET activity within a vessel mask at each timeframe. The vessel mask was defined by segmentation of both external iliac arteries clearly visible on Dixon MR registered to PET. The IDIFs were corrected for spill over of PET activity from surrounding tissue to the arteries and for partial volume errors using the approach described in Croteau et al. [15]. Because the partial volume and spill over corrections were estimated based on measurements made using fluorine-18 and highly dependent on the size of the vessel, the vessel mask was restricted to a segment of each iliac artery where the vessels were relatively straight and had the largest diameter (8–9 mm). The IDIFs were then converted to plasma curves using the average plasma-to-blood ratio determined from the arterial blood sampling. In total, IDIFs were derived for 12 subjects. One subject was excluded due to shorter PET scan, which did not provide data enough for fitting of the method, and two subjects were excluded due to excessive motion, which resulted in difficulties to extract the IDIF. The ratio of the area under the curve (AUC) between IDIF and whole blood AIF (AUCr = AUC_IDIF/AUC_AIF) was calculated to verify equivalency of the two input functions.

### Kinetic analysis and model validation

Kinetic compartmental modeling assumes that the tracer exchanges between blood and tissue compartments by certain rate constants, which can be determined by a set of linear, first-order differential equations. Four compartment models were fitted to the data: an irreversible one-tissue compartment model with rate constant *K*_1_ (1T1k), a reversible one-tissue compartment model with rate constants *K*_1_ and *k*_2_ (1T2k), an irreversible two-tissue compartment model with rate constants *K*_1_, *k*_2_ and *k*_3_ (2T3k) and a reversible two-tissue compartment model with rate constants *K*_1_, *k*_2_, *k*_3_ and *k*_4_ (2T4k). To account for the contribution from blood activity to the tissue TACs, all models were fitted with and without the fractional blood volume *V*_B_. When *V*_B_ was accounted for, it was fitted together with the rate constants. The model that best described the lesion uptake was selected based on the Akaike information criterion (AIC) [16, 17]. The net influx rate *K*_i_ = *K*_1_*k_3_/(*k*_2_+*k*_3_) was calculated from both the irreversible two-tissue compartment model and from the Patlak graphical method [18]. The Patlak model assumes that when all reversible compartments are in equilibrium with plasma, the Patlak plot becomes linear and the positive slope represents the net influx rate *K*_i_. As blood input to the kinetic models, we compared AIF plasma curves and IDIF plasma and whole blood curves. Analysis was done using an in-house MATLAB program (MathWorks, version 2018b).

Simplified protocols and methods for quantification are preferred in routine clinical settings. Therefore, SUV of static images from time-windows 15–30 min, 30–45 min, and 45–60 min were calculated and validated to the net influx rate *K*_i_. SUV and K_i_ were correlated to patient, MR, and histopathological data.

### Statistical analysis

Statistical analysis was done using software R (version 3.4.1, R Core Team (2017)) [19]. The level of significance was set to 5%. The distributions of all numerical variables were visually checked and tested for normality and described accordingly by mean ± standard deviation or by median values with 25th and 75th percentiles (Q1 and Q3). Differences between tissue regions and scan time-windows were tested with the Friedman test. Correlations between patient characteristics, MR parameters, SUV, and kinetic modeling parameters, and between *K*_i_ from the compartment model and graphical Patlak analysis, were assessed using Spearman correlation analysis (rho). For lesion-based analysis, the most avid lesion (highest SUV in the last 15 min of PET scan) per subject was selected to avoid accounting for dependency between multiple lesions from a single subject. The agreement between *K*_i_ using AIF and IDIF was determined using intraclass correlation analysis (ICC3, two-way, mixed model).

## Results

The 15 patients included in the study had a median age of 67 years (Q1 = 62.00, Q3 = 72.50) and a median body weight of 75.00 kg (Q1 = 71.00, Q3 = 84.50) [20]. The median serum PSA level was 8.64 ng/mL (Q1 = 5.31, Q3 = 11.77) with a median PSA density of 0.21 ng/mL/g (Q1 = 0.12, Q3 = 0.43). The mean injected activity per body weight was 2.52 ± 0.67 MBq/kg.

**Fig. 1:**
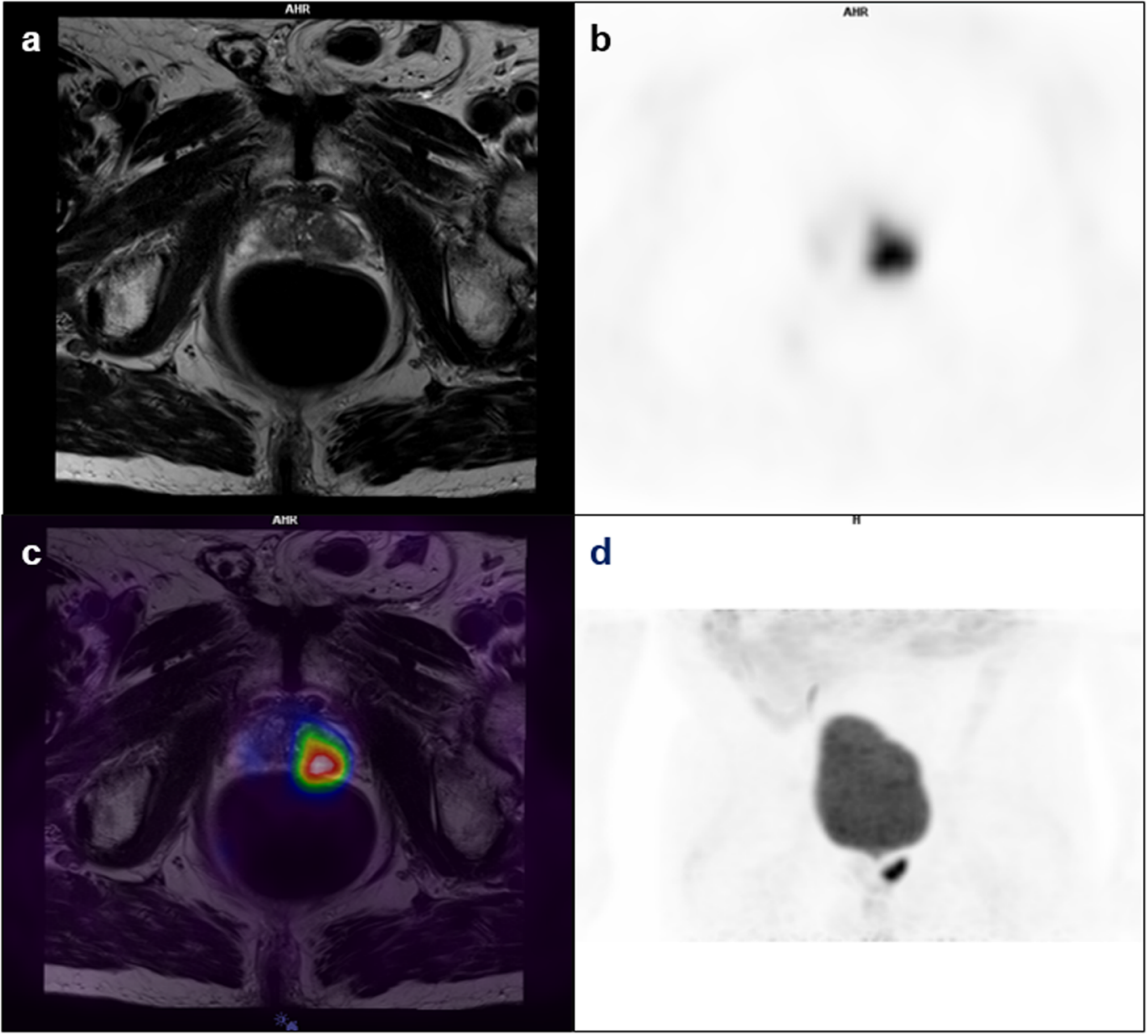
Primary prostate cancer patient (PSA 5.57 ng/mL) presenting an intense focal ^68^Ga-PSMA-11 uptake in the left lobe of the prostate (SUV 45–60 min: 13.27). MR T2-weighted image (**a**), averaged dynamic PET 45–60 min post-injection (**b**), PET/MR fused image (**c**), and whole-body maximum intensity projection of the pelvis (**d**)

One patient had its scan interrupted at 40 min, due to excessive movement. Only lesions identified on both the PET and MR images were considered. One patient was undergoing external beam radiotherapy of the prostate lesion and had no significant ^68^Ga-PSMA-11 uptake. This patient was therefore excluded from lesion-based analysis. Of the 14 ^68^Ga-PSMA-11 positive patients, 10 had 1 lesion and 4 patients had 2 lesions. In total, 18 lesions were identified with a median lesion size of 1.80 cm (Q1 = 1.10, Q3 = 2.75), of which 17 were located in the prostate and 1 identified as a lymph node metastasis. Gleason scores from previous biopsies were between 6 and 10, with a median Gleason score of 7.00 (Q1 = 7.00, Q3 = 7.00). PI-RADS scores for the lesions located in the prostate were between 2 and 5, with a median PI-RADS score of 5.00 (Q1 = 4.00, Q3 = 5.00). The median ADC was 0.71 × 10^−3^ mm^2^/s (Q1 = 0.61 × 10^−3^, Q3 = 0.95 × 10^−3^). Figure 1 shows typical ^68^Ga-PSMA-11 PET/MR images.

**Fig. 2:**
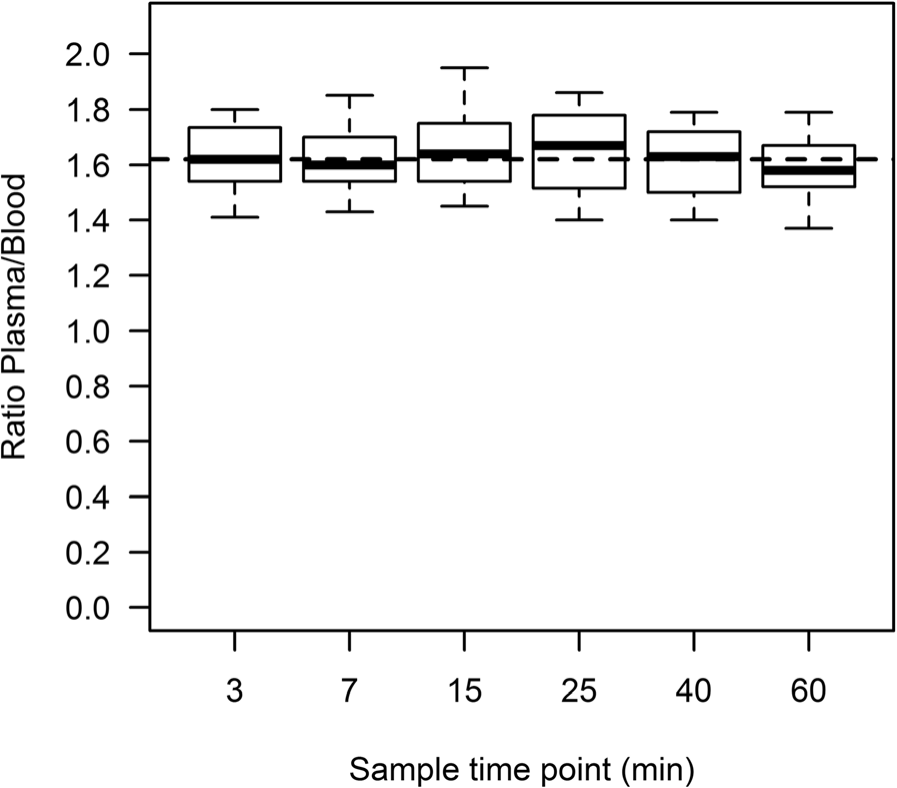
Box plot of the plasma-to-blood ratio over time. The dashed line indicates the overall mean plasma-to-blood ratio of 1.62 (*n* = 83)

**Fig. 3:**
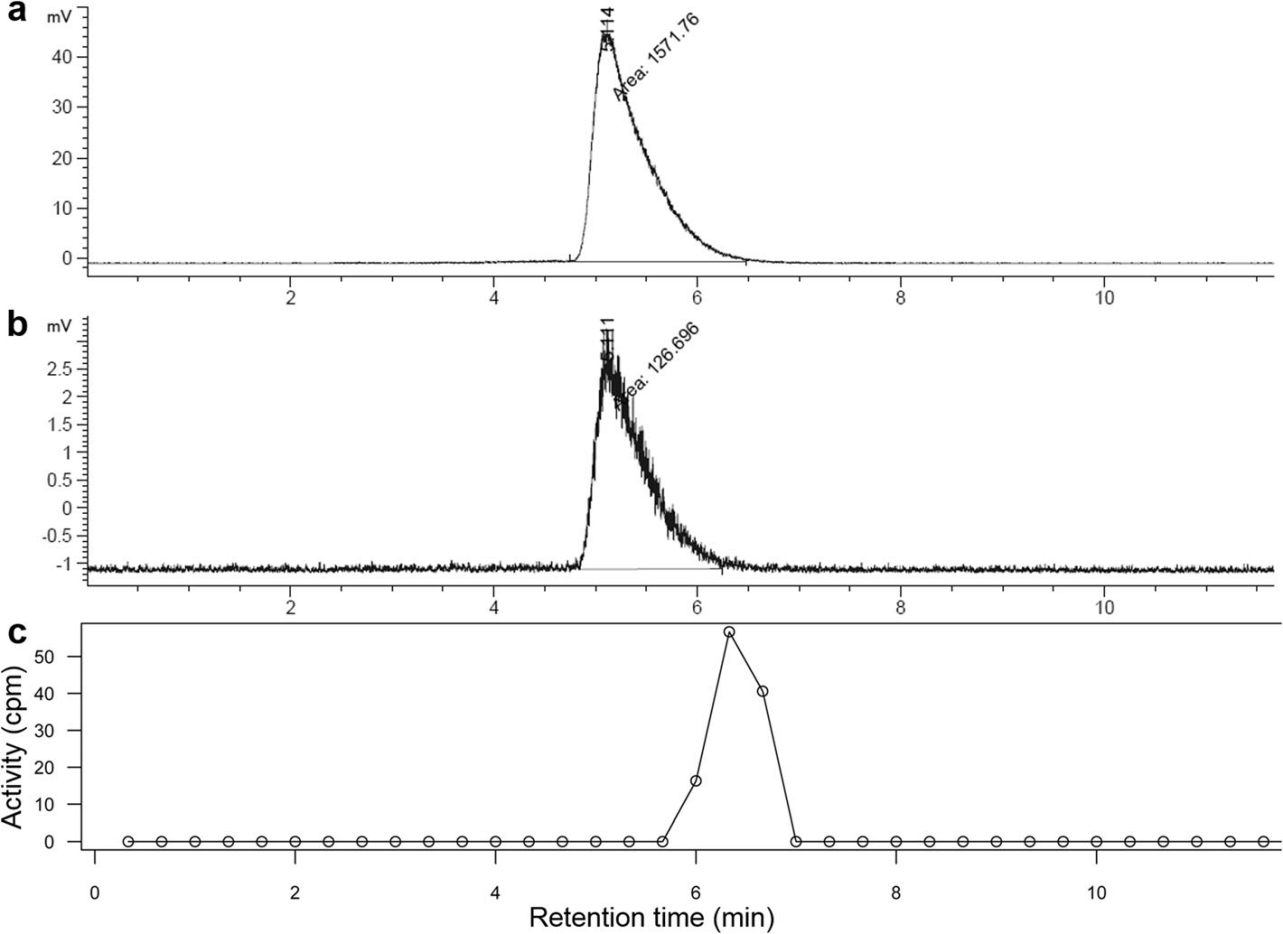
Radiochromatograms from metabolite analysis by UHPLC. Standard solution of ^68^Ga-PSMA-11 with a retention time of 5.1 min (**a**). ^68^Ga-PSMA-11 incubated in plasma in vitro, showing a single peak at 5.1 min (**b**). Patient plasma sample showing a single peak, slightly delayed due to tubing to collect UHPLC fractions (fractions collected every 20 s and subsequently counted in gamma counter) (**c**)

**Fig. 4:**
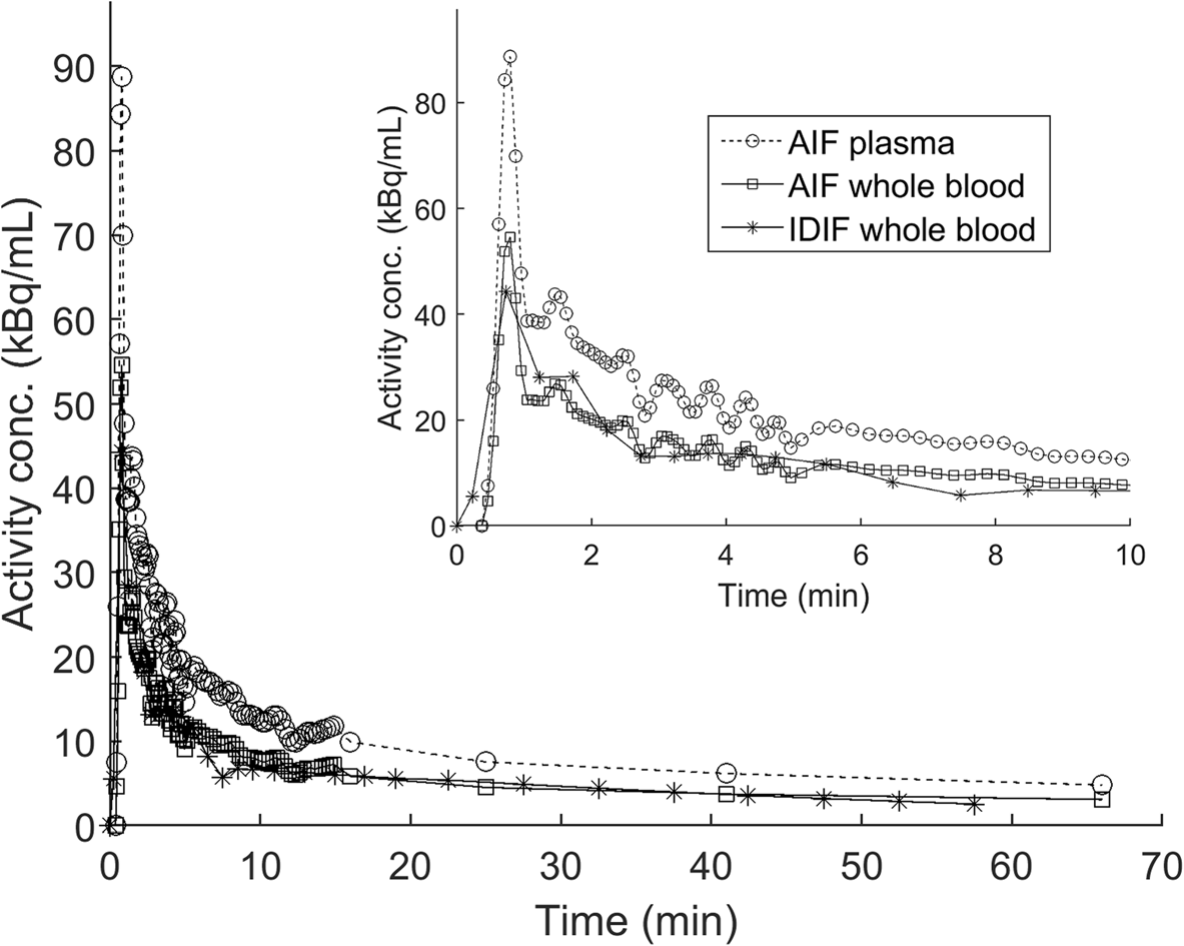
Comparison of typical blood sampler plasma (AIF plasma) and whole blood (AIF whole blood) time-activity curves and corresponding image-derived whole blood curve (IDIF whole blood). Inset graph shows the first 10 min of the blood curves

The activity in whole blood and plasma were counted in 83 manual blood samples. The mean plasma-to-blood ratio was 1.62 and was stable during scan time (Fig. 2). The UHPLC analysis demonstrated that ^68^Ga-PSMA-11 was stable both in vivo and incubated in plasma in vitro, with a retention time of 5.1 min (Fig. 3). Since ^68^Ga-PSMA-11 did not present any radiolabeled metabolites during the 60 min scan time, we could consider the plasma curve as the AIF. Figure 4 shows typical AIF whole blood and plasma curves and IDIF whole blood curve. The mean AUCr between IDIF and AIF curves was 0.84 ± 0.09.

### Kinetic analysis and model validation

**Fig. 5:**
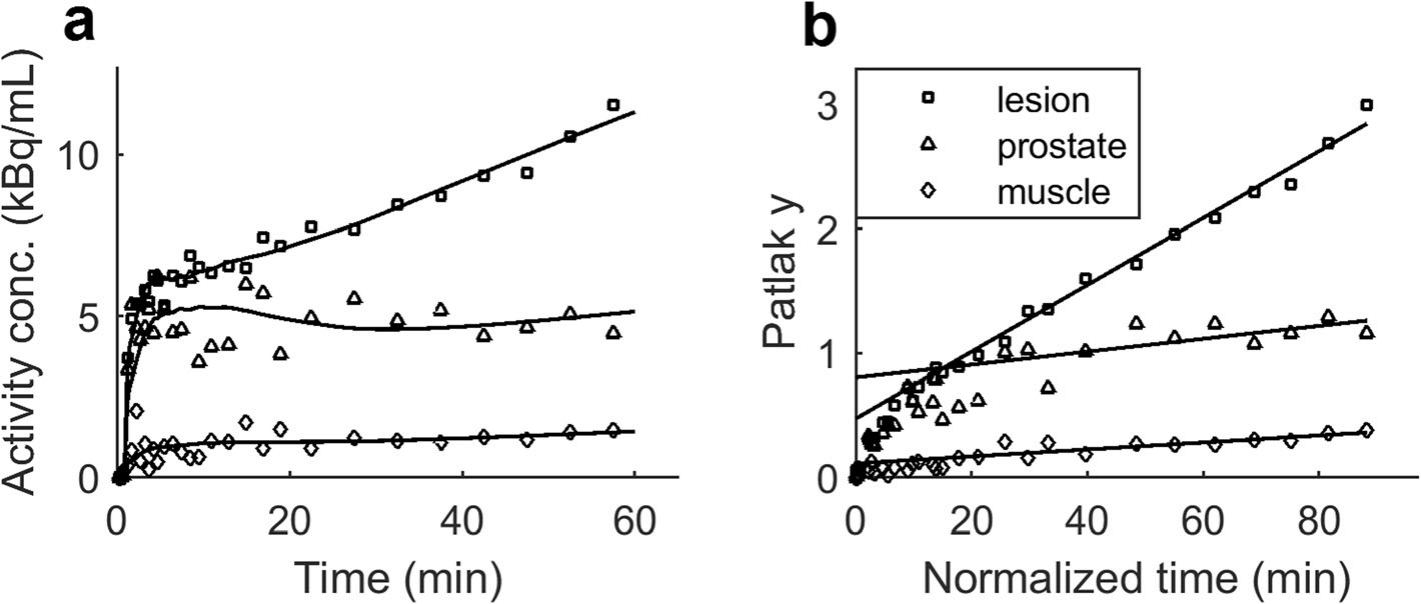
Typical ^68^Ga-PSMA-11 time activity curves and the corresponding irreversible two-tissue compartment model fits (**a**). Patlak graphical analysis presented positive slopes from 15 min post-injection for all regions (**b**)

The tissue TAC’s showed increasing accumulation of ^68^Ga-PSMA-11 during scan time in lesions, while for muscle and normal prostate the washout was faster. Based on the AIC analysis, the irreversible two-tissue compartment model without *V*_B_ correction provided the best fit to the lesion kinetics. The 2T3k model was preferred in 9/18 lesions, followed by the 2T3k + *V*_B_ (3/18), 1T2k (3/18), 2T4k (2/18), and 2T4k + *V*_B_ (1/18). The Patlak graphical analysis showed a positive slope from 15 min post-injection (Fig. 5). Due to movement artifacts at the end of the scan, we excluded the last data points in the model and Patlak fits for five patients. In four lesions, the individual rate constants could not be determined with precision, but *K*_1_ and *K*_i_ as well as Patlak *K*_i_ gave robust values. Rate constants *k*_2_ and *k*_3_ from these lesions were excluded from the median values (Table 1). Both *K*_1_, *k*_3_, *K*_i_ and Patlak *K*_i_ were significantly higher in lesions compared to normal prostate and muscle (Table 1). *K*_i_ from the compartment model analysis and Patlak *K*_i_ showed strong correlation, with rho of 1.00 in lesion (*p* < 0.001), 0.89 in normal prostate (*p* < 0.001) and 0.93 in muscle (*p* < 0.001).

**Table 1:** Kinetic parameters estimated by the irreversible two-tissue-compartment model

	Lesion	Prostate	Muscle	*p* value^a^	*p* value^b^
*K*_1_ (mL/min/mL)	0.136 [0.099, 0.229]	0.088 [0.078, 0.107]	0.028 [0.021, 0.033]	0.006	0.001
*k*_2_ (/mL)	0.277 [0.213, 0.484]	0.239 [0.147, 0.329]	0.285 [0.184, 0.466]	0.308	0.839
*k*_3_ (/mL)	0.108 [0.057, 0.125]	0.040 [0.036, 0.056]	0.040 [0.031, 0.045]	0.005	0.007
*K*_i_ (/mL)	0.036 [0.024, 0.076]	0.014 [0.010, 0.020]	0.003 [0.003, 0.003]	0.001	0.001
Patlak *K*_i_ (/mL)	0.035 [0.024, 0.096]	0.012 [0.008, 0.022]	0.003 [0.003, 0.003]	0.001	0.001

Median values with interquartile range [Q1, Q3], *n* = 14 subjects, 18 lesions (median of *k*_2_ and *k*_3_ from 14 lesions). *p* values assessed by Wilcoxon test (the most avid lesion per subject selected) comparing lesion with prostate (a) and muscle (b). *K*_i_ is the net influx rate calculated from rate constants *K*_1_*k*_3_/(*k*_2_ + *k*_3_) or from the slope of the graphical Patlak analysis

As to the correlation between kinetic rate constants and patient and MR data, we found significant correlation only between *K*_i_ and PSA (rho = 0.62, *p* = 0.018) and between *K*_i_ and PSA density (rho = 0.64, *p* = 0.015). The kinetic rate constants did not correlate significantly with Gleason score, PI-RADS, ADC, or lesion size.

The net influx rate constant *K*_i_ calculated using IDIF plasma showed excellent agreement with *K*_i_ derived using AIF in lesions (*K*_i_ ICC = 0.99, *p* < 0.001 and Patlak *K*_i_ ICC = 0.98, *p* < 0.001), while the individual rate constants *K*_1_ and *k*_3_ showed only moderate agreement with *K*_1_ and *k*_3_ using AIF in lesions (*K*_1_ ICC = 0.57, *p* = 0.026, *k*_3_ ICC = 0.65, *p* = 0.021). IDIF whole blood resulted in slightly weaker agreement with the AIF plasma-derived rate constants (*K*_i_ ICC = 0.89, *p* < 0.001, Patlak *K*_i_ ICC = 0.93, *p* < 0.001).

### Semi-quantitative analysis

SUV increased significantly with time in lesion and muscle, but not in normal prostate and was significantly higher in lesion compared to normal tissue (Table 2).

**Table 2:** ^68^Ga-PSMA-11 SUV compared between regions and time-windows of the PET scan

	Lesion	Prostate	Muscle	*p* value^a^
SUV				
15–30 min	4.95 [3.50, 6.94]	2.48 [2.05, 2.81]	0.54 [0.53, 0.56]	
30–45 min	5.63 [4.04, 9.80]	2.61 [2.15, 3.12]	0.56 [0.52, 0.62]	
45–60 min	5.98 [4.84, 11.72]	2.94 [2.28, 3.49]	0.61 [0.56, 0.72]	<0.001
*p* value^b^	<0.001	0.063	<0.001	

Median values with interquartile range [Q1, Q3], *n* = 14 subjects, 18 lesions. *p* values assessed by Friedman test (the most avid lesion per subject selected) comparing regions (a) and time-windows (b)

Table 3 summarizes the SUV correlation analysis. Lesion SUV of all time-windows correlated significantly with lesion *K*_i_, where SUV of time-window 30–45 min showed the strongest correlation with a rho of 0.95 (*p* < 0.001). SUV correlated significantly with PSA and PSA density, but not with Gleason score, PI-RADS, ADC, or lesion size.

**Table 3:** Correlation analysis for different time-windows of the PET scan

	K_i_		PSA		PSA density	
	Rho	*p* value	Rho	*p* value	Rho	*p* value
SUV						
15–30 min	0.83	< 0.001	0.52	0.056	0.55	0.041
30–45 min	0.95	< 0.001	0.64	0.014	0.61	0.019
45–60 min	0.88	< 0.001	0.65	0.015	0.67	0.012

Spearman correlation rho with corresponding *p* value. *n* = 14, the most avid lesion per subject selected

## Discussion

The main objectives of this study were to determine the kinetics of the PET ligand ^68^Ga-PSMA-11 in primary prostate cancer patients and to validate the use of simplified methods as IDIF and SUV for routine clinical analysis.

Based on the AIC model selection analysis and as indicated by the linear positive Patlak slope, the kinetics of ^68^Ga-PSMA-11 during the 60 min scan time could be considered irreversible, as previous studies have indicated [11, 12]. The irreversible kinetics is in line with the process of internalization of PSMA ligands when binding to the specific binding site of the PSMA transmembrane protein. Internalization of PSMA has been shown to be fast and practically irreversible, as the PSMA ligand is recycled in the lysosomes of the cell [21]. In the compartment model, the internalization can be represented by the rate constant *k*_3_, which as expected was found to be significantly higher in lesion as compared to normal prostate and muscle. *K*_1_, which in the compartment model represents the rate of tracer transport from blood to tissue, was significantly higher in lesions than in normal prostate and muscle, which may reflect the process of angiogenesis in tumor tissue and enhanced tumor tissue permeability [22]. The net influx rate *K*_i_—a macroconstant, which incorporates both the transport from blood to tissue and the irreversible trapping of the tracer—was as expected also significantly higher in lesions as compared to normal prostate and muscle. Sachpekidis et al. [11] reported overall greater absolute values in comparison to our rate constant. This could reflect a more advanced prostate cancer study population: the Sachpekidis’ patient cohort had a higher median serum PSA level of 24.1 ng/mL as compared to ours of 8.6 ng/mL, but similar median age and Gleason score. However, more likely this may be the result of omitting plasma-to-blood ratio correction and the result of partial volume effects due to PET-derived blood input. This would underestimate blood activity and consequently overestimate the kinetic rate constants.

We showed that ^68^Ga-PSMA-11 is stable in vivo, not necessitating correction for labeled metabolites. Due to the lack of metabolites, PSMA makes an ideal candidate for IDIF, only necessitating the correction for plasma-to-blood ratio. With a plasma-to-blood ratio on average 1.62, this indicates that ^68^Ga-PSMA-11 do not penetrate red blood cells and remains in plasma, giving a plasma-to-blood ratio (*R*_P/B_) depending only on hematocrit (HCT) following the equation *R*_P/B_ = 1/(1−HCT). A *R*_P/B_ of 1.62 would equal a HCT factor of 38%, which is within the normal range of HCT for men [23]. As the plasma-to-blood ratio depends only on HCT, the value of 1.62 should be stable between subjects, not necessitating arterial blood sampling in future IDIF studies.

Although IDIF simplifies the acquisition protocol for quantification considerably as compared to blood sampling, for routine clinical settings there is a need for even simpler quantification methods. Here, we showed that SUV of all time-windows correlated significantly with *K*_i_ with correlation coefficients of 0.88 and 0.95 for the late time-windows (Table 3). These are higher than the correlation coefficients of 0.76 for primary and 0.85 for recurrent prostate cancer lesions, reported in previous studies [11, 12]. Again, this might reflect the less accurate PET-derived input function as compared to our arterial input function. We report significant correlation between PSA and SUV (rho = 0.65), similar to the rho of 0.60 reported by Sachpekidis et al. [11], but also a significant correlation between PSA and *K*_i_, differently from Sachpekidis et al. who did not find a significant correlation between the kinetic parameters and PSA. Our data did not, however, present a significant correlation between SUV and Gleason score, differently from Sachpekidis’ study, which reported a weak but significant correlation (rho = 0.33, *p* < 0.05). The lack of correlation between PET and MR parameters might be due to our small patient sample.

The strong correlation between *K*_i_ and SUV demonstrates that SUV can be used to quantify ^68^Ga-PSMA-11 uptake in primary prostate cancer patients. A similar study of the kinetics of [^18^F]fluorocholine concluded that SUV could not be used to quantify [^18^F]fluorocholine uptake due to poor correlation between SUV and *K*_i_ [24], demonstrating the importance of undertaking quantitative studies to validate simplified semi-quantitative methods.

Interestingly, a comparison of the SUV between time-windows 15–30 min, 30–45 min, and 45–60 min showed that the strongest correlation between *K*_i_ and SUV was not seen for the latest time-window, but rather the 30–45 min time-window. This result contrasts from current guidelines and publications, where a standard uptake time of around 60 min, with an acceptable range of 50 and 100 min, is recommended [11, 25–27]. A preliminary analysis of the optimal time-window for lesion visualization showed in agreement with the correlation analysis that the lesions were more easily outlined in early timeframes than in late, mainly due to lower bladder activity in early timeframes. Future studies are needed to determine the optimal time-window for image reporting and quantification.

## Conclusions

The kinetics of ^68^Ga-PSMA-11 are best described by an irreversible two-tissue compartment model. ^68^Ga-PSMA-11 is stable in vivo, which excludes the need for metabolite correction. The net influx rate *K*_i_, estimated using an MR-guided image-derived input function showed excellent agreement with the arterial input function *K*_i_. Image-derived blood can therefore be used as an alternative to arterial blood sampling in kinetic modeling studies. SUV correlated significantly with *K*_i_ and can be used in routine clinical settings to quantify ^68^Ga-PSMA-11 uptake.

## Data Availability

The datasets generated and/or analyzed during the current study are available in the figshare repository, 10.6084/m9.figshare.10265825.

## Abbreviations

1T1k: Irreversible one-tissue compartment model
1T2k: Reversible one-tissue compartment model
2T3k: Irreversible two-tissue compartment model
2T4k: Reversible two-tissue compartment model
ADC: Apparent diffusion coefficients
AIC: Akaike information criterion
AIF: Arterial input function
AUC: Area under the curve
HASTE: Half-Fourier single shot turbo spin echo
HCT: Hematocrit
IDIF: Image-derived input function
MR: Magnetic resonance
OSEM: Ordered-subsets expectation maximization
PET: Positron emission tomography
PI-RADS: Prostate Imaging Reporting and Data System
PSA: Prostate-specific antigen
PSMA: Prostate-specific membrane antigen
PSMA-11: Glu-urea-Lys(Ahx)-HBED-CC
Q1: 25th percentile
Q3: 75th percentile
ROI: Region-of-interest
*R*_P/B_: Plasma-to-blood ratio
SUV: Standardized uptake value
TAC: Time-activity curve
TFA: Trifluoroacetic acid
UHPLC: Ultra-high-pressure liquid chromatography
*V*_B_: Fractional blood volume
VIBE: Volumetric interpolated breath-hold examination
VOI: Volume-of-interest

## References

[1] Evans MJ, Smith-Jones PM, Wongvipat J, Navarro V, Kim S, Bander NH (2011). Noninvasive measurement of androgen receptor signaling with a positron-emitting radiopharmaceutical that targets prostate-specific membrane antigen. Proc Natl Acad Sci U S A.

[2] Afshar-Oromieh A, Zechmann CM, Malcher A, Eder M, Eisenhut M, Linhart HG (2014). Comparison of PET imaging with a (68)Ga-labelled PSMA ligand and (18)F-choline-based PET/CT for the diagnosis of recurrent prostate cancer. Eur J Nucl Med Mol Imaging.

[3] Eiber M, Weirich G, Holzapfel K, Souvatzoglou M, Haller B, Rauscher I (2016). Simultaneous (68)Ga-PSMA HBED-CC PET/MRI improves the localization of primary prostate cancer. Eur Urol.

[4] Jena A, Taneja R, Taneja S, Singh A, Kumar V, Agarwal A (2018). Improving diagnosis of primary prostate cancer with combined (68)Ga-prostate-specific membrane antigen-HBED-CC simultaneous PET and multiparametric MRI and clinical parameters. AJR Am J Roentgenol.

[5] Lutje S, Cohnen J, Gomez B, Gruneisen J, Sawicki L, Rubben H (2017). Integrated (68)Ga-HBED-CC-PSMA-PET/MRI in patients with suspected recurrent prostate cancer. Nuklearmedizin..

[6] Hicks RM, Simko JP, Westphalen AC, Nguyen HG, Greene KL, Zhang L (2018). Diagnostic accuracy of (68)Ga-PSMA-11 PET/MRI compared with multiparametric MRI in the detection of prostate cancer. Radiology..

[7] Tomasi G, Turkheimer F, Aboagye E (2012). Importance of quantification for the analysis of PET data in oncology: review of current methods and trends for the future. Mol Imaging Biol.

[8] Feng ST, Cui M, Gao J, Wu B, Sha W, Huang B (2012). Image-derived arterial input function in dynamic positron emission tomography-computed tomography: a method using both positron emission tomographic and computed tomographic images. J Comput Assist Tomogr.

[9] Anazodo U, Kewin M, Finger E, Thiessen J, Hadway J, Butler J (2015). Preliminary evaluation of MRI-derived input function for quantitative measurement of glucose metabolism in an integrated PET-MRI. EJNMMI Phys.

[10] Pfob CH, Ziegler S, Graner FP, Kohner M, Schachoff S, Blechert B (2016). Biodistribution and radiation dosimetry of (68)Ga-PSMA HBED CC-a PSMA specific probe for PET imaging of prostate cancer. Eur J Nucl Med Mol Imaging.

[11] Sachpekidis C, Kopka K, Eder M, Hadaschik BA, Freitag MT, Pan L (2016). 68Ga-PSMA-11 Dynamic PET/CT Imaging in Primary Prostate Cancer. Clin Nucl Med.

[12] Sachpekidis C, Eder M, Kopka K, Mier W, Hadaschik BA, Haberkorn U (2016). (68)Ga-PSMA-11 dynamic PET/CT imaging in biochemical relapse of prostate cancer. Eur J Nucl Med Mol Imaging.

[13] Eder M, Schafer M, Bauder-Wust U, Hull WE, Wangler C, Mier W (2012). 68Ga-complex lipophilicity and the targeting property of a urea-based PSMA inhibitor for PET imaging. Bioconjug Chem.

[14] Weinreb JC, Barentsz JO, Choyke PL, Cornud F, Haider MA, Macura KJ (2016). PI-RADS prostate imaging-reporting and data system: 2015, Version 2. Eur Urol.

[15] Croteau E, Lavallee E, Labbe SM, Hubert L, Pifferi F, Rousseau JA (2010). Image-derived input function in dynamic human PET/CT: methodology and validation with 11C-acetate and 18F-fluorothioheptadecanoic acid in muscle and 18F-fluorodeoxyglucose in brain. Eur J Nucl Med Mol Imaging.

[16] Akaike H (1974). A new look at the statistical model identification. IEEE Trans Autom Control.

[17] Golla SSV, Adriaanse SM, Yaqub M, Windhorst AD, Lammertsma AA, van Berckel BNM (2017). Model selection criteria for dynamic brain PET studies. EJNMMI Phys.

[18] Patlak CS, Blasberg RG, Fenstermacher JD (1983). Graphical evaluation of blood-to-brain transfer constants from multiple-time uptake data. J Cereb Blood Flow Metab.

[19] R Core Team. R: A language and environment for statistical computing. Vienna, Austria: R Foundation for Statistical Computing; 2017.

[20] Ringheim A, Campos Neto G De C, Anazodo UC, Cui L, da Cunha ML, Vitor T, et al.. Pharmacokinetic modeling of 68Ga-PSMA-11 in primary prostate cancer patients [Internet]. figshare; 2019 [cited 2019Nov25].. Available from: https://figshare.com/articles/Pharmacokinetic_modeling_of_68Ga_PSMA-11_in_primary_prostate_cancer_patients/10265825/2

[21] Ghosh A, Heston WD (2004). Tumor target prostate specific membrane antigen (PSMA) and its regulation in prostate cancer. J Cell Biochem.

[22] Luczynska E, Aniol J (2013). Neoangiogenesis in prostate cancer. Contemporary oncology (Poznan, Poland).

[23] National Heart, Lung, and Blood Institute: Types of blood tests. http://www.nhlbi.nih.gov/health/health-topics/topics/bdt/types. Accessed 31 Oct 2019.

[24] Verwer EE, Oprea-Lager DE, van den Eertwegh AJ, van Moorselaar RJ, Windhorst AD, Schwarte LA (2015). Quantification of 18F-fluorocholine kinetics in patients with prostate cancer. J Nucl Med.

[25] Afshar-Oromieh A, Malcher A, Eder M, Eisenhut M, Linhart HG, Hadaschik BA (2013). PET imaging with a [68Ga]gallium-labelled PSMA ligand for the diagnosis of prostate cancer: biodistribution in humans and first evaluation of tumour lesions. Eur J Nucl Med Mol Imaging.

[26] Fendler Wolfgang P., Eiber Matthias, Beheshti Mohsen, Bomanji Jamshed, Ceci Francesco, Cho Steven, Giesel Frederik, Haberkorn Uwe, Hope Thomas A., Kopka Klaus, Krause Bernd J., Mottaghy Felix M., Schöder Heiko, Sunderland John, Wan Simon, Wester Hans-Jürgen, Fanti Stefano, Herrmann Ken (2017). 68Ga-PSMA PET/CT: Joint EANM and SNMMI procedure guideline for prostate cancer imaging: version 1.0. European Journal of Nuclear Medicine and Molecular Imaging.

[27] Afshar-Oromieh A, Hetzheim H, Kubler W, Kratochwil C, Giesel FL, Hope TA (2016). Radiation dosimetry of (68)Ga-PSMA-11 (HBED-CC) and preliminary evaluation of optimal imaging timing. Eur J Nucl Med Mol Imaging.

